# Fluorescent visualization of oxytocin in the hypothalamo-neurohypophysial system

**DOI:** 10.3389/fnins.2014.00213

**Published:** 2014-07-23

**Authors:** Hirofumi Hashimoto, Takanori Matsuura, Yoichi Ueta

**Affiliations:** Department of Physiology, School of Medicine, University of Occupational and Environmental HealthKitakyushu, Japan

**Keywords:** CCK, c-fos, double transgenic rat, mRFP1, paraventricular nucleus, posterior pituitary, supraoptic nucleus

## Abstract

Oxytocin (OXT) is well known for its ability to the milk ejection reflex and uterine contraction. It is also involved in several other behaviors, such as anti-nociception, anxiety, feeding, social recognition, and stress responses. OXT is synthesized in the magnocellular neurosecretory cells (MNCs) in the hypothalamic paraventricular (PVN) and the supraoptic nuclei (SON) that terminate their axons in the posterior pituitary (PP). We generated transgenic rats that express the OXT and fluorescent protein fusion gene in order to visualize OXT in the hypothalamo-neurohypophysial system (HNS). In these transgenic rats, fluorescent proteins were observed in the MNCs and axon terminals in the PP. This transgenic rat is a new tool to study the physiological role of OXT in the HNS.

## Introduction

Oxytocin (OXT), a nine amino acid neuropeptide, was discovered in 1906 as an extract with uterus-contracting effects from the pituitary (Dale, 1906). In 1953, OXT was the first peptide hormone to be sequenced and synthesized (du Vigneaud et al., 1953a,b, 1954). OXT is synthesized primarily in magnocellular neurosecretory cells (MNC) in the hypothalamic paraventricular (PVN) and the supraoptic nuclei (SON), which cells project their axon terminals into the posterior pituitary (PP), where it is released into the systemic circulation, in the same way as arginine vasopressin (AVP). OXT is well known for its roles in reproduction, especially during and after childbirth. Many previous studies have shown that OXT is involved in several physiological functions, such as antinociception, anxiety, feeding, social recognition, and stress responses (Carmichael et al., 1987, 1994; Stock and Uvnäs-Moberg, 1988; Uvnäs-Moberg et al., 1993; Leckman et al., 1994; Russell and Leng, 1998).

We recently have reported the generation and characterization of rats which faithfully express an AVP-enhanced green fluorescent protein (eGFP) fusion transgene (Ueta et al., 2005; Fujio et al., 2006; Shibata et al., 2007; Suzuki et al., 2009; Maruyama et al., 2010; Todoroki et al., 2010; Iwanaga et al., 2011; Ohno et al., 2012). Previous studies that used animals to examine OXT dynamics by fluorescent visualization reported about OXT-enhanced cyan fluorescent protein (eCFP) transgenic mice (Young et al., 1999; Zhang et al., 2002). Although we first generated an OXT-eCFP transgenic rat, the expression of the transgene was unstable for unknown reasons (Katoh et al., 2010). Monomeric red fluorescent protein (mRFP) was developed from DsRed, which is the red fluorescent protein from *Discosoma* (Campbell et al., 2002; Long et al., 2005), and we succeeded in generating transgenic rats bearing an OXT-mRFP1 fusion gene (Katoh et al., 2011).

In this review, we focus on (1) the distribution of OXT, (2) the regulation of synthesis and release of OXT, and (3) recent research about the visualization of OXT in OXT transgenic animals using a fluorescent protein, which is a new tool to study the physiological role of OXT in the hypothalamo-neurohypophysial system (HNS).

## Distribution of oxytocin and oxytocin receptor

In MNC in the PVN and the SON, OXT neurons project their axon terminals into the PP. In parvocellular neurosecretory cells (PNC) in the PVN, OXT neurons project their axon terminals to the spinal code, including the intermediolateral nucleus and gelatinous substance, where OXT have some role to modify pains and sympathetic nervous system (Sofroniew, 1980). OXT cells also project to the ambiguus nucleus, the nuclei of solitary tract (NTS), the dorsal motor nucleus of vagus, the Edinger–Westphal nucleus, circularis nucleus (CN), the parabrachial nucleus, the hippocampus, the amygdaloid nucleus, and the septulum (Reaves and Hayward, 1979; Nilaver et al., 1980; Sofroniew, 1980; Sofroniew et al., 1981; Hatton and Tweedle, 1982; Sawchenko and Swanson, 1982). Parvocellular OXT cells are found in the preoptic area and the lateral hypothalamus, whereas accessory magnocellular OXT cells are found scattered across the hypothalamus.

The central effects of OXT are mediated by OTRs distributed widely in the brain. OTR mRNAs are distributed in the ventromedial nucleus of the hypothalamus (VMH) and the PVN, which are involved in steroid-sensitive reproductive behaviors; in the substantia nigra and ventral tegmental area, which is involved in maternal behaviors; in the hippocampus, which is involved in learning and memory; and in the lateral septum, caudate putamen, amygdaloid nuclei, olfactory tubercle and cingulate, perirhinal, and frontal cortices, all of which are involved in reinforcement (Ostrowski, 1998).

## Regulation of synthesis and release of oxytocin

OXT is produced in the MNC of the PVN and the SON, and is released into the systemic circulation from axon terminals in the neurohypophysis, particularly during parturition, lactation and in response to osmotic challenges (Burbach et al., 2006). The structure of the OXT gene was elucidated in 1984 (Ivell and Richter, 1984). Expression of the OXT gene is stimulated during pregnancy and lactation (Van Tol et al., 1988; Zingg and Lefebvre, 1988). Interestingly, although estrogen or progesterone alone does not increase OXT synthesis expression of the OXT gene in the PVN and the SON was increased by the prolonged administration of estrogen and progesterone, followed by progesterone withdrawal (Thomas and Amico, 1996). By contrast, OXT gene expression in the uterine was highly stimulated by the combined application of estrogen and progesterone (Lefebvre et al., 1994).

OXT is well known for its roles in reproduction, especially during and after childbirth. The pulsatile OXT release into the circulation is stimulated by vaginocervical stimulation associated with labor and the stimulus of suckling on the nipple. The uterine muscle increases its OXT receptors (OTRs) and sensitivity to OXT during the latter few months of pregnancy. That level of OXT release from the neurohypophysis is considerably increased at the time of labor. In lactation, OXT causes milk to be expressed from the alveoli into the ducts of the breast so that the baby can obtain it by suckling. The suckling stimulus on the nipple of the breast causes signals to be transmitted through sensory nerves to the OXT, secreting neurons in the MNC in the PVN and the SON. OXT in plasma is carried to the breast, where it causes contraction of myoepithelial cells that lie outside of and form a latticework surrounding the alveoli of the mammary glands. In less than a minute after a baby starts suckling, milk begins to flow.

The sequence of the OTR was reported in 1992 (Kimura et al., 1992; Kubota et al., 1996). Gonadal steroids play an important role in mediating the regulation of OTR expression. Most peripheral OXT-binding sites, including the pituitary, renal, and uterine, are upregulated by estrogens (Fuchs et al., 1983; Soloff et al., 1983; Maggi et al., 1992). The upregulation is accompanied by OTR mRNA expression, suggesting that the upregulation is a consequence of a genomic estrogen effect on the OTR gene transcription (Breton et al., 1995; Larcher et al., 1995). Behavioral studies have clearly shown that a necessary potential of OXT to elicit maternal or sexual behavior is priming with estrogen alone or with both estrogen and progesterone (Pedersen et al., 1982; Fahrbach et al., 1985). This evidence suggests that OTRs are under the control of gonadal steroids in the central nervous system (CNS).

OTR gene expression increases during pregnancy and/or at parturition in the olfactory bulb, medial preoptic area, bed nucleus of the stria terminalis (BNST), the SON, and in the medial amygdala in rat (Young et al., 1997; Meddle et al., 2007). Studies have shown that OTR-binding sites increase in the medial preoptic area, the BNST, VMH, and the ventral tegmental area on postpartum day 1 (Insel, 1990; Pedersen et al., 1994; Young et al., 1997). These changes suggest that OXT and OTR receptors play a role in both lactation and the regulation of maternal behavior.

OXT is also recognized as having endocrine and paracrine roles in male reproduction. OXT is synthesized within the mammalian testis, epididymis and prostate, and OTRs in the reproductive tract support a local action for OXT (Ivell et al., 1990, 1997; Foo et al., 1991; Nicholson and Hardy, 1992; Frayne and Nicholson, 1995, 1998; Harris et al., 1996; Filippi et al., 2002; Whittington et al., 2004). In ejaculation, a burst of OXT is released from the neurohypophysis into the systemic circulation and stimulates contractions of the reproductive tract for sperm release (Ogawa et al., 1980; Carmichael et al., 1987; Murphy et al., 1987). OXT plays a paracrine role in stimulating contractility of the seminiferous tubules, epididymis and the prostate gland.

Interestingly, OXT is also released from soma and dendrites during parturition and lactation (Ludwig and Leng, 2006). Although OXT released from the soma and dendrites of the MNC in the SON and the PVN may act in a paracrine to activate distant receptors (Ludwig and Leng, 2006), OXT-like immunoreactivity (LI) fibers can be found throughout the brain, including the nucleus accumbens (NAcc), lateral septum, amygdala, and some areas in the hindbrain, brainstem, and spinal cord (Sofroniew, 1980; Castel and Morris, 1988). A notable reduction of OXT-LI fibers was observed throughout the brain by the lesioning of the PVN (De Vries and Buijs, 1983). Although little is known about the regulation of OXT release from these forebrain projections, they might contribute significantly to the regulation of behavior.

## Transgenic animal of oxytocin

### OXT deficient mice

Previous studies have generated mice carrying a deletion of the OXT-coding region using homologous recombination in embryonic stem cells (Nishimori et al., 1996; Young et al., 1996). Mice lacking OXT are both viable and fertile. Males do not have any reproduction behavioral or functional defects in the absence of OXT. Similarly, females have no obvious deficits in fertility or reproduction, including gestation and parturition. Although OXT-deficient females demonstrated normal maternal behavior, all their offspring died of starvation shortly after birth, because OXT-deficient mothers were unable to nurse. After injections of OXT to OXT-deficient mothers, milk ejection was induced and the offspring survived. OXT-deficient male mice fail to develop social memory (Ferguson et al., 2000). A measurement of both olfactory foraging and olfactory habituation tasks has indicated that olfactory detection of non-social stimuli is intact in OXT-deficient male mice, and treatment with OXT reinstates social memory in those mice. These data indicate that OXT is necessary for the normal development of social memory in mice and support the hypothesis that social memory has a neural basis distinct from other forms of memory.

### OXTR deficient mice

OXTR-deficient mice were viable and had no obvious deficits in fertility or reproductive behavior, the same as OXT-deficient mice (Takayanagi et al., 2005). OXTR-deficient dams mice exhibited normal parturition but demonstrated defects in lactation and maternal nurturing. Infant OXTR-deficient males emitted fewer ultrasonic vocalizations than their wild-type littermates in response to social isolation. Adult OXTR-deficient males also showed deficits in social discrimination, and demonstrated increased aggressive behavior. OXT-deficient males from OXT-deficient but not from heterozygote dams showed high levels of aggression. These data suggest a developmental role for the OXT/OXTR system in shaping adult aggressive behavior.

### Animals bearing fluorescent fusion transgenes

Previous studies have shown the placement of the eGFP coding sequence (Young et al., 1999; Zhang et al., 2002) or chloramphenicol acetyltransferase (CAT) reporters at various locations within an OXT transgene (Jeong et al., 2001) (Table 1). We generated rats bearing an OXT-eCFP fusion transgene designed from a murine construct previously shown to be faithfully expressed in transgenic mice (Katoh et al., 2010) (Table 1). However, the expression of the transgene was unstable for unknown reasons.

**Table 1:** **Oxytocin transgenes**.

**Transgenesis**		**Transgene**	**Reporter gene**	**Specificity expression in HNS**	**Ectopic expression**	**References**
**From**	**To**					
Mouse	Mouse	AI-02	eGFP	None	None	Young et al., 1999
Mouse	Mouse	AI-01	eGFP	Few	None	Young et al., 1999
Mouse	Mouse	AI-03	eGFP	+	None	Young et al., 1999
Mouse	Mouse	JL-01	IRES-eGFP	+	None	Young et al., 1999
Mouse	Mouse	OT-3-CAT-3.5	CAT	+	None	Jeong et al., 2001
Mouse	Rat	AI-03	eCFP	+	None	Katoh et al., 2010
Rat	Rat	OXT-mRFP1	mRFP1	+	None	Katoh et al., 2011, 2014

*HNS, hypothalamo-neurohypophysial system*.

**Figure 1:**
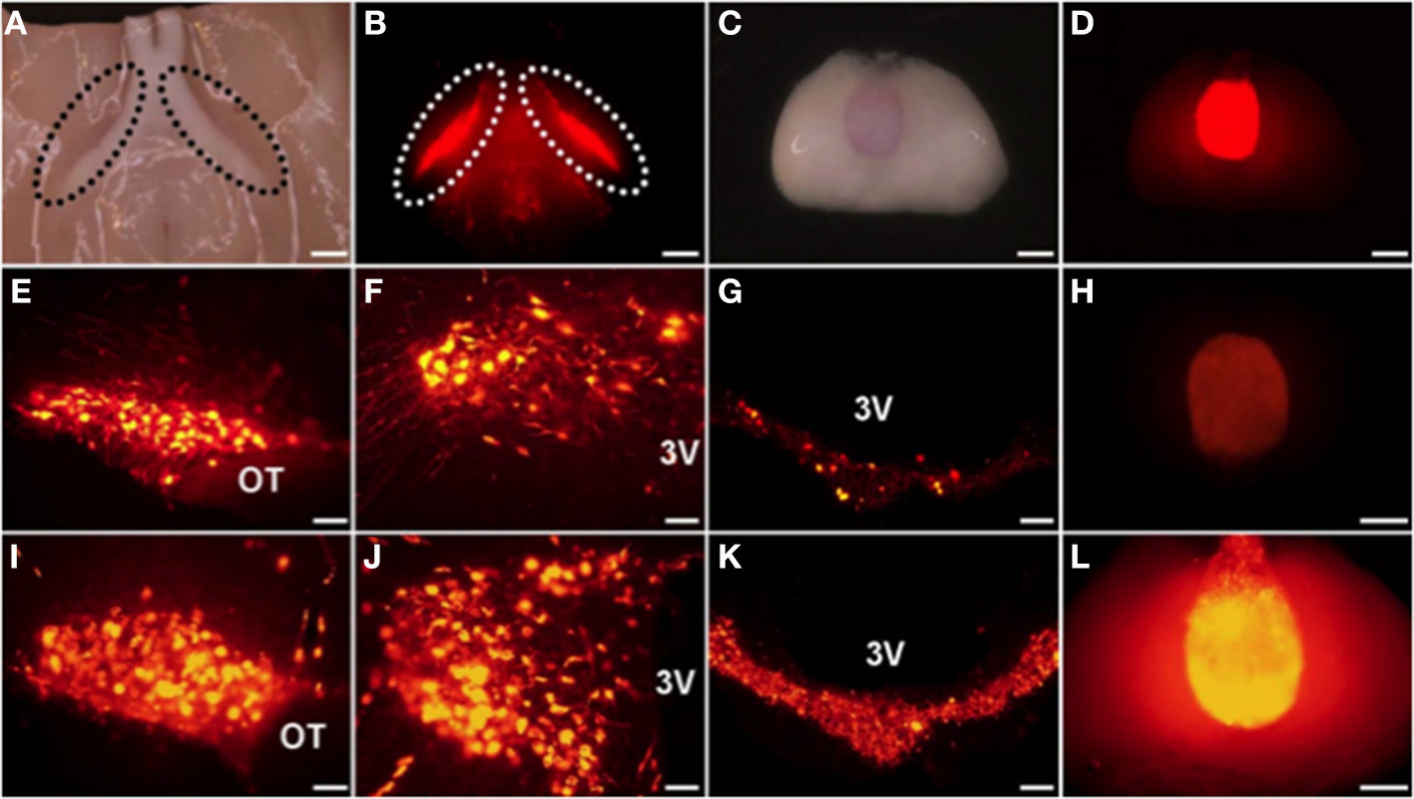
**The mRFP1 fluorescence was clearly observed in ventral parts of the supraoptic nucleus (SON) (A,B) and in the PP (C,D) without cutting**. Endogenous florescence of mRFP1 in the SON **(E)**, the paraventricular nucleus (PVN) **(F)**, the median eminence (ME) **(G)**, and the posterior pituitary (PP) **(H)**. Effects of salt loading for 5 days on the mRFP1 fluorescence of the SON **(I)**, the PVN **(J)**, the ME **(K)**, and the PP **(L)**. Under light **(A,C)** and fluorescent **(B,D–L)**. *Scale bars*, 1 mm **(A–D,H,L)** and 0.1 mm **(E–G,I–K)**. OT, Optic tract; 3V, third ventricle. Modified with permission from Figure 1 in Katoh et al. (2011).

The mRFP was developed from DsRed, which is the red fluorescent protein from *Discosoma* (Campbell et al., 2002; Long et al., 2005). We have succeeded in generating transgenic rats bearing an OXT-mRFP1 fusion gene (Katoh et al., 2011) (Table 1) (Figure 1). Interestingly, when the brains of these rats were mounted on a slide, the mRFP1 fluorescence was visible in the ventral part of the SON and in the PP without cutting. We could observe the mRFP1 fluorescence throughout the SON, especially in the dorsal parts. We could observe abundant mRFP1 fluorescence in the magnocellular division of the PVN and scattered mRFP1 fluorescence in the parvocellular division of the PVN. We also observed mRFP1 fluorescence in the internal layer of the median eminence (ME) and in the PP. *In situ* hybridization histochemistry showed mRFP1 mRNA localized in the SON and in the magnocellular and parvocellular divisions of the PVN. In comparing male and female transgenic rats under normal conditions, there were no differences in the expression of mRFP1 mRNA in the SON and the PVN. In comparing nontransgenic and transgenic rats under normal conditions, there were no differences between them in plasma osmorality, sodium, OXT, AVP, and the expression of the endogenous OXT gene and AVP gene in the SON and the PVN.

Previous studies have reported that OXT transcripts significantly increased in the rat hypothalamus after chronic osmotic stimuli, such as salt loading (Lightman and Young, 1987; McCabe et al., 1990; Yue et al., 2008). In our OXT-mRFP1 transgenic rats, the fluorescence of mRFP1 was remarkably increased by 5 to 7-fold throughout the SON and in the PVN, ME, and PP after salt loading for 5 days (Katoh et al., 2010) (Figure 1). *In situ* hybridization histochemistry showed dramatically increased the expression of the mRFP1 mRNA in the SON and the PVN after salt loading. Comparing nontransgenic and transgenic rats after salt loading, there were no differences in plasma osmorality, sodium, OXT, AVP, and the expression of the endogenous OXT gene and AVP gene in the SON and the PVN.

**Figure 2:**
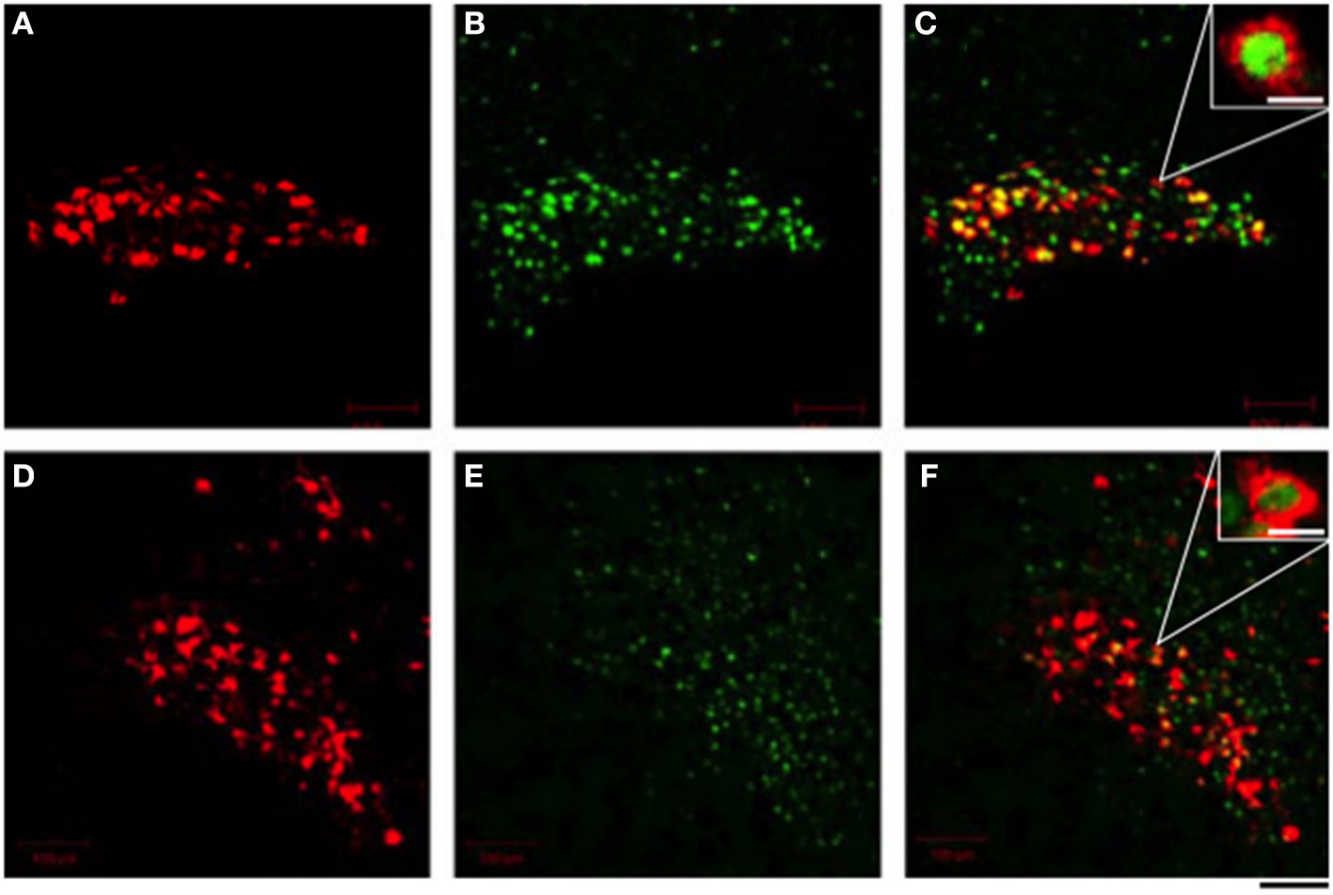
**Effects of i.p. administration of cholecystokinin-8 on the endogenous fluorescence of monomeric red fluorescent protein 1 (mRFP1) (A,D) and nuclear enhanced green fluorescent protein (eGFP) (B,E) in the supraoptic nucleus (A–C) and the paraventricular nucleus (D–F)**. The merged view of fluorescence of mRFP1 and eGFP was seen as a yellow color **(C,F)**. Scale bars shown in white represent 10 μm in **(C,F)**. The scale bar shown in black = 40 μm. Modified with permission from Figure 3 in Katoh et al. (2014).

The peripheral administration of cholecystokinin (CCK) -8 stimulated secretion of OXT but not AVP (Verbalis et al., 1986; Ueta et al., 2000; Hashimoto et al., 2005), and excited OXT-secreting magnocellular neurons in the SON and the PVN in rats (Hamamura et al., 1991; Ueta et al., 1993, 2000; Hashimoto et al., 2005). CCK-8 stimulates gastric vagal afferents and activated noradrenergic neurons in the nucleus of the tractus solitarius (Luckman, 1992). It is postulated that these noradrenergic inputs activate OXT-secreting neurons in the SON and the PVN and cause the secretion of OXT into the systemic circulation in rats (Hamamura et al., 1991). Recently, we have developed a novel transgenic rat that enables the trivial visualization of c-*fos* expression using an eGFP tag (Katoh et al., 2014). These rats express a transgene consisting of c-*fos* gene regulatory sequences that drive the expression of a c-*fos*-eGFP fusion protein. Moreover, we generated a double transgenic rat that expresses both the c-*fos*-eGFP and an OXT-mRFP1 fusion gene. In these double transgenic rats, nuclear eGFP fluorescence appeared in OXT-mRFP1 neurons in the SON and the PVN 90 min after i.p. administration of CCK-8 (Figure 2). Three-dimensional reconstruction imaging enables the visualization of nuclear eGFP in the cytoplasm of OXT neurons illuminated and identified by virtue of their expression of mRFP1. In these neurons, abundant OXT granules in the cytoplasm are clearly visible by a plane image obtained from a higher magnification by confocal laser microscopy (Katoh et al., 2014).

## Conclusions

We did not observe any fluorescence of mRFP1 in the ectopic area of OXT in the OXT-mRFP1 transgenic rats. The OXT neuron has the same proper response to physiological stimulation in the OXT-mRFP1 transgenic rats as in nontransgenic rats. Using OXT-mRFP1 rats, we can identify the OXT neuron easily and see changes in the neuron's activity and release of OXT in realtime. Moreover, we can see smaller changes that we had not been able to see before, because OXT-mRFP1 transcription is more sensitive than endogenous OXT transcription to the same stimulation. The OXT-mRFP1 transgenic rats are a useful animal model to study dynamic changes in OXT in the HNS.

### Conflict of interest statement

The authors declare that the research was conducted in the absence of any commercial or financial relationships that could be construed as a potential conflict of interest.
